# Multisite functional connectivity MRI classification of autism: ABIDE results

**DOI:** 10.3389/fnhum.2013.00599

**Published:** 2013-09-25

**Authors:** Jared A. Nielsen, Brandon A. Zielinski, P. Thomas Fletcher, Andrew L. Alexander, Nicholas Lange, Erin D. Bigler, Janet E. Lainhart, Jeffrey S. Anderson

**Affiliations:** ^1^Interdepartmental Program in Neuroscience, University of UtahSalt Lake City, UT, USA; ^2^Department of Psychiatry, University of UtahSalt Lake City, UT, USA; ^3^Departments of Pediatrics and Neurology, University of Utah and Primary Children's Medical CenterSalt Lake City, UT, USA; ^4^School of Computing and Scientific Computing and Imaging Institute, University of UtahSalt Lake City, UT, USA; ^5^Waisman Laboratory for Brain Imaging and Behavior, Department of Psychiatry, University of WisconsinMadison, WI, USA; ^6^Department of Medical Physics, University of WisconsinMadison, WI, USA; ^7^Departments of Psychiatry and Biostatistics, Harvard UniversityBoston, MA, USA; ^8^Neurostatistics Laboratory, McLean HospitalBelmont, MA, USA; ^9^Department of Psychology and Neuroscience Center, Brigham Young UniversityProvo, UT, USA; ^10^The Brain Institute of Utah, University of UtahSalt Lake City, UT, USA; ^11^Department of Bioengineering, University of UtahSalt Lake City, UT, USA; ^12^Division of Neuroradiology, University of UtahSalt Lake City, UT, USA

**Keywords:** functional connectivity, fcMRI, classification, autism, ABIDE

## Abstract

**Background**: Systematic differences in functional connectivity MRI metrics have been consistently observed in autism, with predominantly decreased cortico-cortical connectivity. Previous attempts at single subject classification in high-functioning autism using whole brain point-to-point functional connectivity have yielded about 80% accurate classification of autism vs. control subjects across a wide age range. We attempted to replicate the method and results using the Autism Brain Imaging Data Exchange (ABIDE) including resting state fMRI data obtained from 964 subjects and 16 separate international sites.

**Methods**: For each of 964 subjects, we obtained pairwise functional connectivity measurements from a lattice of 7266 regions of interest covering the gray matter (26.4 million “connections”) after preprocessing that included motion and slice timing correction, coregistration to an anatomic image, normalization to standard space, and voxelwise removal by regression of motion parameters, soft tissue, CSF, and white matter signals. Connections were grouped into multiple bins, and a leave-one-out classifier was evaluated on connections comprising each set of bins. Age, age-squared, gender, handedness, and site were included as covariates for the classifier.

**Results**: Classification accuracy significantly outperformed chance but was much lower for multisite prediction than for previous single site results. As high as 60% accuracy was obtained for whole brain classification, with the best accuracy from connections involving regions of the default mode network, parahippocampaland fusiform gyri, insula, Wernicke Area, and intraparietal sulcus. The classifier score was related to symptom severity, social function, daily living skills, and verbal IQ. Classification accuracy was significantly higher for sites with longer BOLD imaging times.

**Conclusions**: Multisite functional connectivity classification of autism outperformed chance using a simple leave-one-out classifier, but exhibited poorer accuracy than for single site results. Attempts to use multisite classifiers will likely require improved classification algorithms, longer BOLD imaging times, and standardized acquisition parameters for possible future clinical utility.

## Introduction

Brain imagingclassification strategies of autism have used information from structural MRI (Ecker et al., 2010a,b; Jiao et al., 2010; Uddin et al., 2011; Calderoni et al., 2012; Sato et al., 2013), functional MRI (Anderson et al., 2011d; Coutanche et al., 2011; Wang et al., 2012), diffusion tensor MRI (Lange et al., 2010; Ingalhalikar et al., 2011), positron emission tomography (Duchesnay et al., 2011), and magnetoencephalography (Roberts et al., 2010, 2011; Tsiaras et al., 2011; Khan et al., 2013). Such approaches have been undertaken for several clinical objectives. Sensitive and specific biomarkers for autism may contribute potentially useful biological information to diagnosis, prognosis, and treatment decision-making. It is hoped that imaging biomarkers may also help delineate subtypes of individuals with autism that may have common brain neuropathology and respond to similar treatment strategies, although different methodology will likely be required for subgrouping individuals than for classifying individuals by diagnosis. Such quantitative biomarkers may also serve as a metric for biological efficacy of potential behavioral or pharmacologic interventions. Finally, imaging biomarkers may help identify pathophysiologic mechanisms of autism in the brain that can guide investigations into the specific neural circuits, developmental windows, and genetic or environmental factors that may result in improved treatments.

Abnormal functional connectivity MRI (fcMRI) has been among the most replicated imaging metrics in autism. The proposed basis for fcMRI is that connected brain regions are likely to exhibit synchronized neural activity, which can be detected as covariance of slow fluctuations in Blood Oxygen Level Dependent (BOLD) signal between the regions. Initial reports of decreased functional connectivity in autism by three independent groups (Just et al., 2004; Villalobos et al., 2005; Welchew et al., 2005) have been followed by more than 50 primary reports of abnormal functional connectivity in autism in the literature, derived from fMRI data both in a resting state and acquired during cognitive tasks (Anderson, 2013).

Most reports show decreases in connectivity between distant brain regions, including nodes of the brain's default mode network (Cherkassky et al., 2006; Kennedy and Courchesne, 2008; Wiggins et al., 2011), social brain regions (Gotts et al., 2012; von dem Hagen et al., 2013), attentional regions (Koshino et al., 2005), language regions (Dinstein et al., 2011), interhemispheric homologues (Anderson et al., 2011a), and throughout the brain (Anderson et al., 2011d). Nevertheless, some reports have also shown abnormal increases in functional connectivity in autism (Muller et al., 2011) or unchanged connectivity (Tyszka et al., 2013). In particular, higher correlation between brain regions has been observed in negatively correlated connections (Anderson et al., 2011d), corticostriatal connections (Di Martino et al., 2011), visual search regions (Keehn et al., 2013), and brain network-level metrics (Anderson et al., 2013a; Lynch et al., 2013).

Despite the large and growing body of reports of abnormal functional connectivity in autism, uncertainty remains about the spatial distribution of decreased and increased connectivity and how this relates to the clinical heterogeneity of autism spectrum disorders (ASD). One of the challenges for answering these questions has been fractionation of the available data into individual site-specific studies with relatively small sample sizes. There is a need for analysis of multisite datasets that can improve statistical power, represent greater variance of disease and control samples, and allow replication across multiple sites with differential subject recruitment, imaging parameters, and analysis methods. Ultimately, clinically useful biomarkers will need to be replicated in diverse acquisition conditions that reflect community and academic imaging practices.

The advent of cooperative, publicly available datasets for resting state functional MRI is an important step forward. Multiple such datasets have now been released including the 1000 functional connectome project (Biswal et al., 2010), the ADHD 200 Consortium dataset (ADHD-200_Consortium, 2012), and most recently the Autism Brain Imaging Data Exchange (ABIDE) (Di Martino et al., 2013), consisting of images from 539 individuals with ASD and 573 typical control individuals, acquired at 16 international sites. In the present study, we evaluate classification accuracy of whole-brain functional connectivity across sites, and determine which abnormalities in connectivity across the brain are most informative for predicting autism from typical development, which imaging acquisition features lead to greatest accuracy, whether functional connectivity abnormalities covary with metrics of disease severity, and the extent to which abnormal functional connectivity is replicated across sites.

## Materials and methods

### Subject sample

ABIDE consists of 1112 datasets comprised of 539 autism and 573 typically developing individuals (Di Martino et al., 2013). Each dataset consists of one or more resting fMRI acquisitions and a volumetric MPRAGE image. All data are fully anonymized in accordance with HIPAA guidelines, with analyses performed in accordance with pre-approved procedures by the University of Utah Institutional Review Board. All images were obtained with informed consent according to procedures established by human subjects research boards at each participating institution. Details of acquisition, informed consent, and site-specific protocols are available at fcon_1000.projects.nitrc.org/indi/abide/.

Inclusion criteria for subjects were successful preprocessing with manual visual inspection of normalization to MNI space of MPRAGE, coregistration of BOLD and MPRAGE images, segmentation of MPRAGE image, and full brain coverage from MNI *z* > −35 to *z* < 70 on all BOLD images. Inclusion criteria for sites were a total of at least 20 subjects meeting all other inclusion criteria. A total of 964 subjects met all inclusion criteria (517 typically developing subjects and 447 subjects with autism from 16 sites). Each site followed different criteria for diagnosing patients with autism or ascertaining typical development, however, the majority of the sites used the Autism Diagnostic Observation Schedule (Lord et al., 2000) and Autism Diagnostic Interview-Revised (Lord et al., 1994). Specific diagnostic criteria for each site can be found at fcon_1000.projects.nitrc.org/indi/abide/index.html.

Subject demographics for individuals satisfying inclusion criteria are shown in Table 1. Six different testing batteries were used to calculate verbal IQ and performance IQ, respectively. In addition to the IQ measures, the following measures were included in correlations with the classifier score (see Table 1 for summary of behavioral measures):the Social Responsiveness Scale (Constantino et al., 2003) is a measure of social function and the Vineland Adaptive Behavior Scales (Sparrow et al., 1984) is a measure of daily functioning. See the ABIDE website for more information on the specific behavioral measures used. For handedness, categorical handedness (i.e., right-handed, left-handed, or ambidextrous) was used in the leave-one-out classifier (see details below). In the case that only a quantitative handedness measure was reported, positive values were converted to right-handed, negative values to left-handed, and a value of zero to ambidextrous. Fifteen subjects lacked a categorical and quantitative measure of handedness. In those cases, a nearest neighbor classification function (ClassificationKNN.m in MATLAB) was used to assign categorical handedness. For the classifier, 862 subjects were right-handed, 95 were left-handed, and 7 were ambidextrous.

**Table 1:** **Subjects included from the ABIDE sample with demographic information**.

	**Age**	**ADI-R social**	**ADI-R verbal**	**ADOS total**	**Verbal IQ**	**Performance IQ**	**SRS total**	**Vineland**
Number of subjects	964	348	349	348	781	796	335	201
Control	(426 M, 91 F)	0	0	32	413	425	160	80
Autism	(396 M, 51 F)	348	349	316	367	371	175	121
Control mean ±*SD*	16.9 ± 7.56	NA	NA	1.25 ± 1.37	112 ± 13.3	108 ± 13.3	21.2 ± 16.2	105 ± 11.6
(Control range)	(6.47–56.2)	NA	NA	(0–4)	(67–147)	(67–155)	(0–103)	(77–131)
Autism mean ±*SD*	16.6 ± 8.1	19.7 ± 5.65	15.9 ± 4.55	11.9 ± 3.81	105 ± 17.4	106 ± 17.2	91.6 ± 30.6	75 ± 13.2
(Autism range)	(7–64)	(2–30)	(2–26)	(2–22)	(50–149)	(59–157)	(6–164)	(41–106)

### BOLD preprocessing

Preprocessing was performed in MATLAB (Mathworks, Natick, MA) using SPM8 (Wellcome Trust, London) software. The following sequence of preprocessing steps was performed:

1. Slice timing correction.
2. Realign and reslice correction of motion for each volume relative to initial volume.
3. Coregistration of BOLD images to MPRAGE anatomic sequence.
4. Normalization of MPRAGE to MNI template brain, with normalization transformation also applied to coregistered BOLD images.
5. Segmentation of gray matter, white matter, and CSF components of MPRAGE image (thorough clean).
6. Voxelwisebandpass filter (0.001–0.1 Hz) and linear detrend
  - [(a)] The lower limit of 0.001 Hz was chosen in order to be certain as much neural information was included as possible (Anderson et al., 2013b). The linear detrend removed much of the contribution of low frequencies given the relatively short time series available in the dataset.
7. Extraction of mean time courses from the restriction masks applied to BOLD images from ROIs consisting of:
  - CSF segmented mask with bounding box −35 < *x* < 35, −60 < *y* < 30, 0 < *z* < 30.
  - White matter segmented mask overlapping with 10 mm radii spheres centered at *x* = −27, *y* = −7, *z* = 30, *x* = 27, *y* = −7, *z* = 30.
  - Mask of scalp and facial soft tissues (Anderson et al., 2011b).
8. Voxelwise regression using glmfit.m (MATLAB Statistics Toolbox) software of CSF, WM, Soft tissue, and 6 motion parameters from realignment step from time series of each voxel of BOLD images.
9. Motion scrubbing (Power et al., 2012) of framewise displacement and DVARS with removal of volumes before and after a root-mean-square displacement of >0.2 mm for either parameter and concatenation of remaining volumes. In 86.2% of the participants more than 50% of the volumes remained after motion scrubbing. Among the remaining participants with fewer than 50% retained volumes, the majority belonged to the autism group (8.8%, compared to 5.0% from the typically developing group; *p* = 0.02). The groups differed in the number of retained volumes when considering the entire sample of 964 subjects (*t* = 4.11, *p* < 0.001) and when considering only those with greater than 50% of the volumes remaining (*t* = 2.04, *p* = 0.04).
10. No spatial smoothing was performed. The global mean signal and gray matter time courses were not regressed from voxelwise data (Saad et al., 2012, 2013; Jo et al., 2013).

### ROI analysis

From preprocessed BOLD images for each subject, mean time course was extracted from 7266 gray matter ROIs. These ROIs from a lattice covering the gray.nii image (SPM8) from *z* = −35 to *z* = 70 at 5-mm resolution, with MNI coordinates of centroids previously reported (Anderson et al., 2011d). The ROIs averaged 4.9 ± 1.3 standard deviation voxels in size for 3 mm isotropic voxels. A 7266 × 7266 matrix of Fisher-transformed Pearson correlation coefficients was obtained for each subject from the ROI timecourses representing an association matrix of functional connectivity in each subject between all pairs of ROIs. Each pair of ROIs is termed a “connection” for the present analysis.

### Leave-one-out classifier

**Figure 1:**
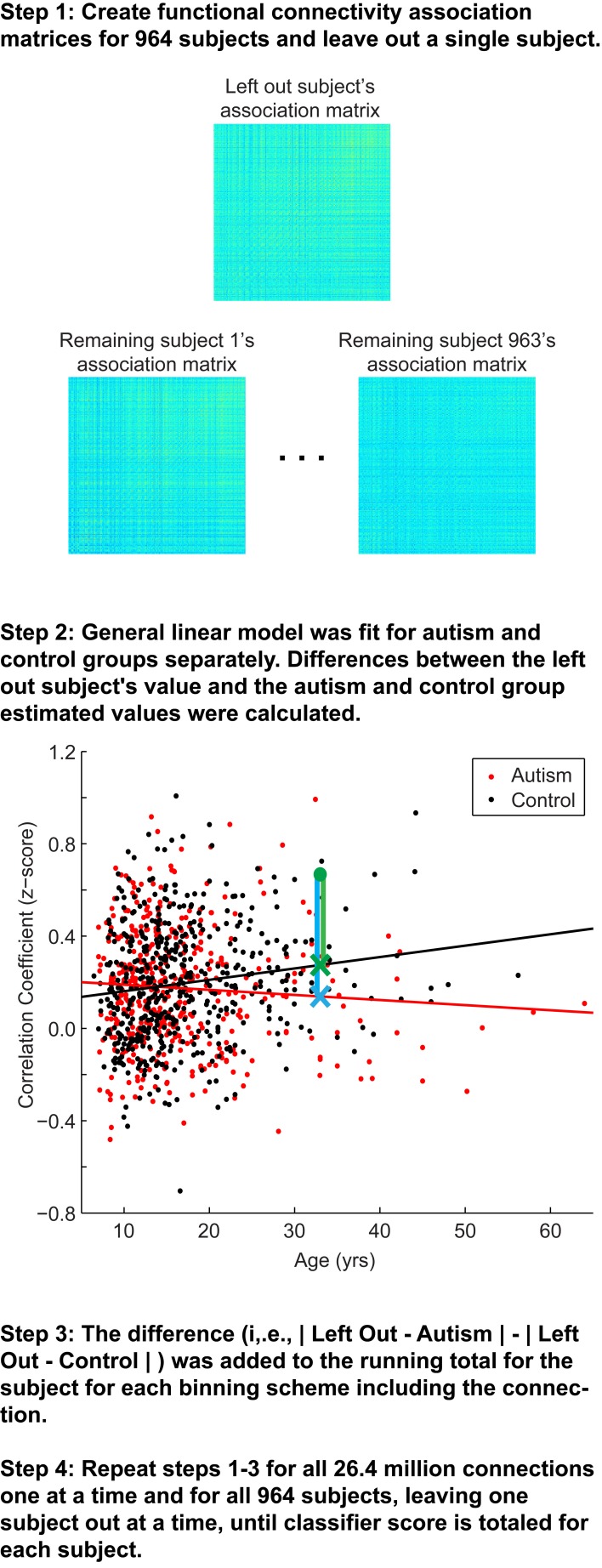
**Summary of classification approach**. Step 1, Association matrices corresponding to the intrinsic connectivity between each pair of 7266 gray matter regions (about 26.4 million connections) are estimated for the left out subject and the 963 remaining subjects. Step 2, Plot depicting an example connection (i.e., single cell of the possible 26.4 million cells from the association matrices in Step 1) for the 964 subjects. The plot includes axes for correlation strength and age, however, the plot represents a multidimensional space that includes age-squared, gender, and handedness as covariates. *Black line*, fit line for the control group; *red line*, fit line for the autism group; *green data point*, left out subject (a control subject in this example); *green X*, estimated value for the control group; *blue X*, estimated value for autism group; *green vertical line*, difference between actual connection strength value for left out subject and estimated value for control group; *blue vertical line*, difference between actual connection strength value for left out subject and estimated value for autism group. Steps 3 and 4 are described in the text.

The classification approach is summarized in Figure 1. Overall, a leave-one-out classifier was used to generate a classification score for each of the 964 subjects, leaving out one subject at a time and calculating the classification score for the left out subject. The classification approach followed the approach reported previously, with slight modifications (Anderson et al., 2011d). First, the correlation measurements for the remaining 963 subjects were extracted for one of the 26.4 million connections from the 7266 × 7266 association matrix described above (Figure 1, Step 1). Second, a general linear model was fit to the measurements separately for autism (red fit line in Figure 1, Step 2) and control subjects (black fit line in Figure 1, Step 2) for the given connection with covariates of subject age, age-squared, gender, and handedness. From these data, estimated values for the left out subject for this connection were calculated based on the left out subject's age, gender, and handedness. A value was estimated separately from the remaining autism subjects (blue X in Figure 1, Step 2) and remaining control subjects (green X in Figure 1, Step 2).

Because each site used slightly different scanning hardware and parameters that may systematically bias results, the estimated values of the left out subject (blue and green X in Figure 1, Step 2) were adjusted by adding the difference of the site's mean value for that connection (minus the left out subject) from the mean value for that connection from all other sites. Finally, the actual value for the left out subject for the connection (green dot in Figure 1, Step 2) was subtracted from the estimated value obtained from autism subjects (blue vertical line on Figure 1, Step 2) and from the estimated value obtained from control subjects (green vertical line in Figure 1, Step 2). The difference of the absolute value of these two differences was then multiplied by the F-statistic for the difference between the remaining autism and control subjects. This process was iteratively carried out for all 26.4 million connections and then averaged across the 7265 connections in which each of 7266 ROIs participates. Then the averaged values for each of the 7266 ROIs were summed. The summed value was equal to the classification score for the subject. More negative values for the classification score predict the left out subject was a control subject, and more positive values for classification score predict the left-out subject was an autism subject.

### Bins of “connections”

Connections were grouped into bins in several different ways to aggregate groups of connections to test for accuracy in discriminating autism from control subjects. First, a measurement of correlation strength was obtained for each connection from 961 independent subjects from the 1000 Functional Connectome project using identical preprocessing steps (see y-axis of Figure 6). Subjects included in this sample have been previously described (Ferguson and Anderson, 2011). Second, Euclidean distance between each pair of ROIs was calculated from the centroid coordinates for the ROIs (see x-axis of Figure 6). Connections were grouped into 2-dimensional bins based on the strength of the correlation and the distance between the ROIs, with bin spacing of 0.05 units of Fisher-transformed correlation and 5-mm distance. The results for accurately classifying the subjects using this binning system are summarized in Figure 6.

A separate binning scheme was performed during the evaluation of a leave-one-out-classifier. For each left out subject, sets of connections were calculated that satisfied a two-tailed *t*-test between remaining autism and control subjects with *p*-values less than 0.01, 0.001, 0.0001, and 0.00001. These sets of connections varied slightly for each left out subject, since no data that can reflect the value of the left-out subject's connectivity measurement can be used in the classifier.

Classification accuracy, sensitivity, and specificity were calculated for the set of connections that differed between autism and control subjects at *p*-values of 0.01, 0.001, 0.0001, 0.00001 (Figure 3A). We used this last binning system because there is a tradeoff in using many connections in constructing the classifier scores and using fewer but more informative connections. We wanted to determine which thresholded bin yielded the highest accuracy.

### Statistical analyses

For each bin of connections, a vector of 964 classification scores was obtained (one for each left out subject) and the classification score was thresholded at 0 (in the case of the strength/Euclidean distance bins, or at a threshold selected to optimize the area under a receiver operating characteristic curve for the case of the bins determined by *p*-values. Predicted diagnosis (autism vs. control) was compared to the actual diagnosis of each left out subject, and significant classification accuracy was determined by a binomial distribution. For 964 subjects, predicting 509 subjects (52.8%) correctly corresponded to an uncorrected *p*-value of less than 0.05, and predicting 531 subjects (55.1%) correctly corresponds to *p*-value of less than 0.001. Two-proportion *z*-tests were used to test the following: (1) whether there was a group difference in the proportion of subjects with less than 50% of the BOLD volumes remaining after motion scrubbing (results above in *BOLD preprocessing section*), (2) whether classification accuracy differed between the eyes open and eyes closed subjects, (3) whether classification accuracy differed between the male and female subjects, and (4) whether accuracy increased when considering only those subjects with greater than 50% of the BOLD volumes remaining after motion scrubbing, rather than all 964 subjects. Two-sample *t*-tests were used to determine if there was a group difference in the number of remaining volumes (results above in *BOLD preprocessing section*).

## Results

**Figure 2:**
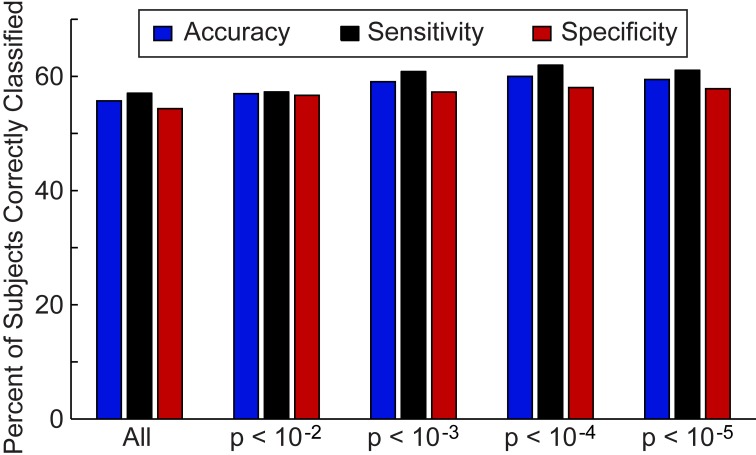
**Total accuracy, sensitivity, and specificity for leave-one-out classifier in 964 subjects**. The total accuracy, sensitivity, and specificity are shown when all 26.4 million connections were included in the classifier and then for different *p*-value thresholds that determine which connections are included in the classifier.

**Figure 3:**
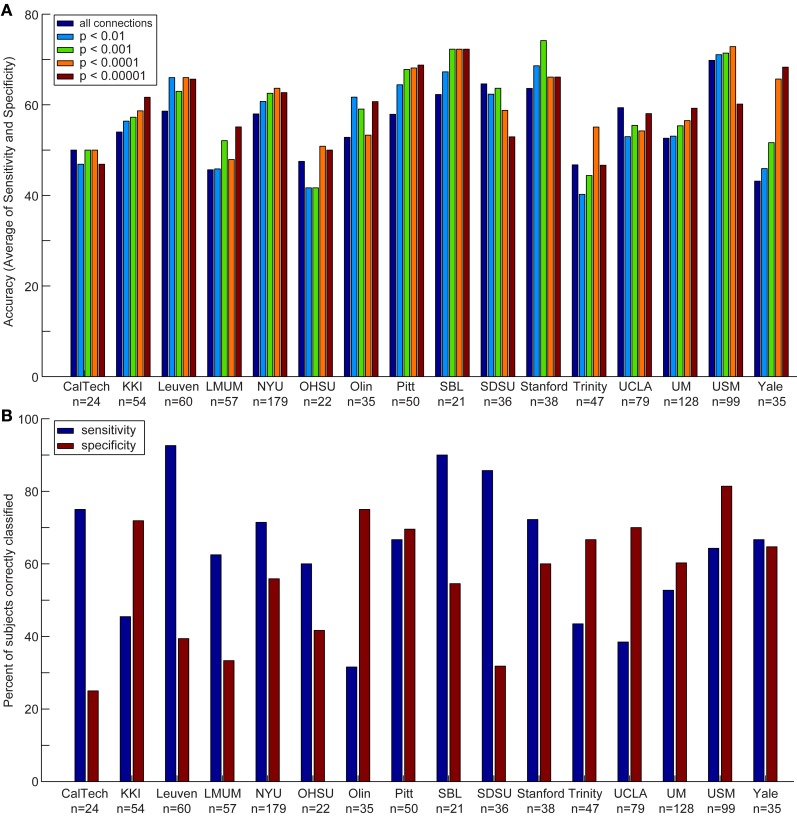
**Accuracy, sensitivity, and specificity for each data acquisition site**. Accuracy **(A)** is shown for each data acquisition site at different *p*-value thresholds. The sensitivity and specificity **(B)** are shown for each data acquisition site at a threshold of *p* < 0.0001 (i.e., the threshold at which optimal total accuracy was obtained in Figure 2).

First, we investigated the overall accuracy, sensitivity, and specificity of the leave-one-out classifier for all 964 subjects in the ABIDE consortium (Figure 2) and the 16 data collection sites individually (Figure 3). For the entire ABIDE consortium, we achieved the highest overall accuracy (60.0%), sensitivity (62.0%), and specificity (58.0%) when connections were included in the classification algorithm if group differences for the connection met a *p*-value threshold of less than 10^−4^; whereas the lowest accuracy (55.7%), sensitivity (57.1%), and specificity (54.4%) were found when all 26.4 million connections were included in the leave-one out classifier. When considering only those subjects with greater than 50% of the BOLD volumes remaining after motion scrubbing, the accuracy for the five different *p*-value thresholds increased between 0.6% and 3.1%, although the difference was not significant compared to the accuracy for all 964 subjects (*p* > 0.18). No difference in classification accuracy was found between subjects who had their eyes open during the scan vs. those who had their eyes closed, after correcting for multiple comparisons using an FDR of *q* < 0.05. Also, no difference in classification accuracy was found between male and female subjects, after correcting for multiple comparisons using an FDR of *q* < 0.05.

We also compared the accuracy, sensitivity, and specificity across sites using different *p*-value thresholds for determining which connections to include in the leave-one-out classifier. The accuracy, sensitivity, and specificity varied at each site depending on the *p*-value threshold, however, we consistently achieved the highest accuracy at SBL (mean accuracy = 69.3%), USM (mean accuracy = 69.1%), Stanford (mean accuracy = 67.7%), and Pitt (mean accuracy = 65.4%); the highest sensitivity at SDSU (90.0%), Leuven (88.9%), SBL (84.0%), and Stanford (74.4%); and the highest specificity at USM (79.5%), Olin (75.0%), UCLA (71.5%), and KKI (70.6%).

**Figure 4:**
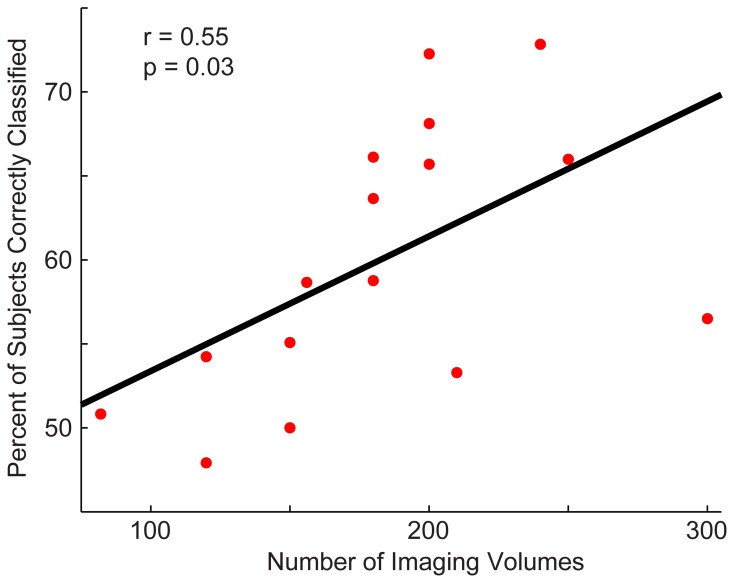
**Relationship between a site's total accuracy and the number of imaging volumes acquired by each site**. Each site's total accuracy was calculated when using a *p* < 0.0001 threshold (i.e., the threshold at which optimal total accuracy was obtained in Figure 2) and correlated with the number of BOLD imaging volumes acquired during the resting-state sequence.

Next, we determined whether the site's sample size or the number of imaging volumes from a single run related to the site's classification accuracy (Figure 4). The number of imaging volumes was positively correlated with accuracy (*r* = 0.55, *p* = 0.03). If the number of imaging volumes post-scrubbing was averaged across site, the relationship between number of imaging volumes and accuracy was no longer significant. Sample size did not correlate with site's classification accuracy (*r* = 0.17, *p* = 0.53).

**Figure 5:**
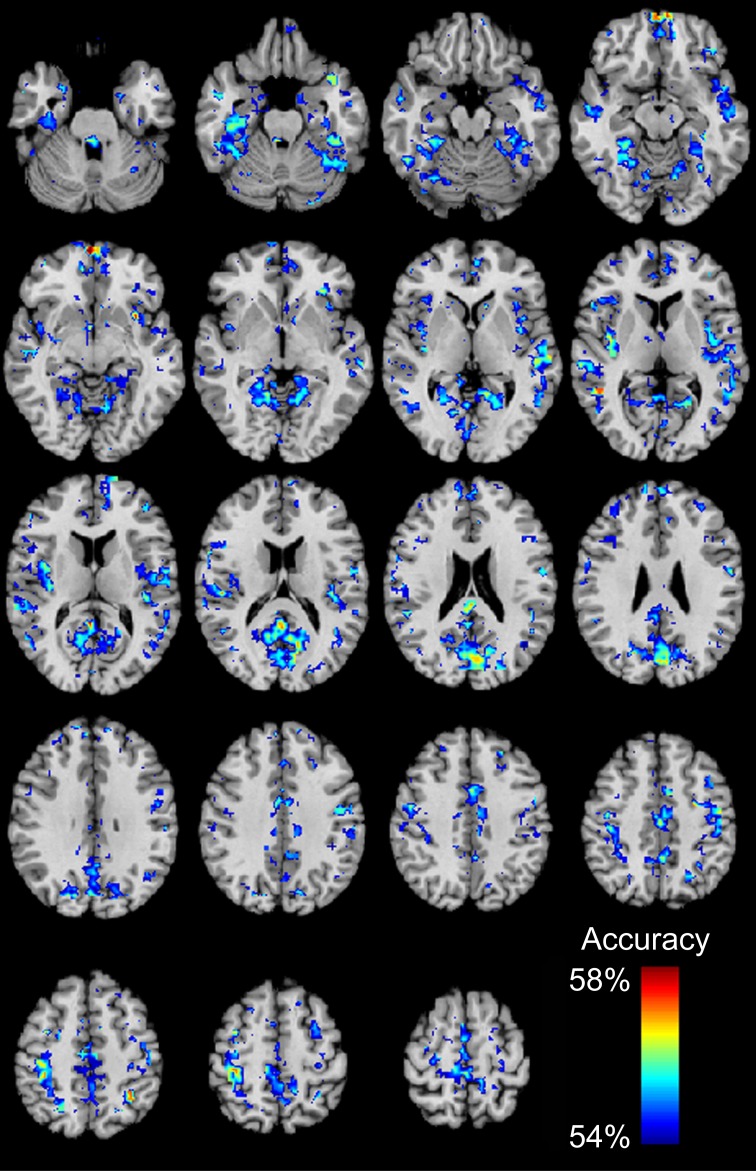
**Total accuracy for 7266 brain regions**. Accuracy was determined for each of the 7266 brain regions independently by only taking into account the 7265 connections in which a given region was involved (no *p*-value threshold, all connections used). The minimum accuracy displayed for a single region is 53.95%, which was the false discovery rate corrected percentage for 7266 regions and a binomial cumulative distribution.

**Figure 6:**
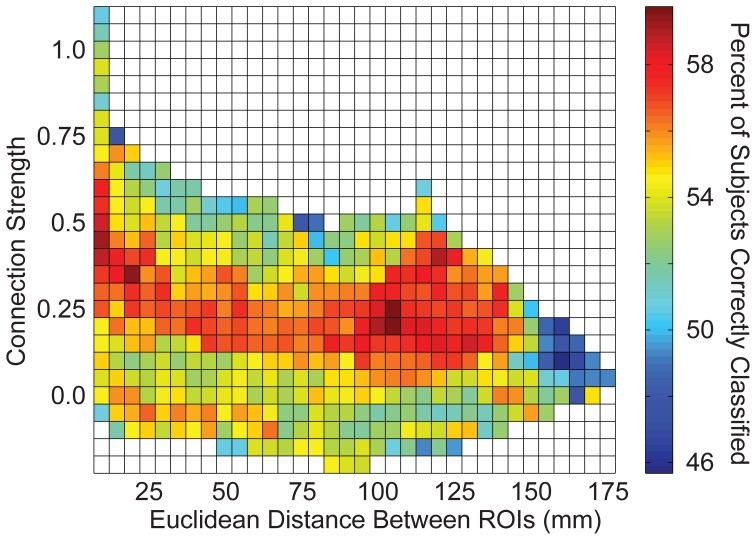
**Total accuracy across connection strength and distance between brain regions**. The 26.4 million connections were divided up into bins based on the correlation strength of the connection (determined by an independent sample) and the distance between the connection's two endpoints. Accuracy is displayed for each bin with at least one connection.

We then determined which brain regions and connection characteristics accurately classified the ABIDE subjects. In Figure 5, the following brain regions (and the 7265 connections in which they were involved) resulted in the highest accuracy: parahippocampaland fusiform gyri, insula, medial prefrontal cortex, posterior cingulate cortex, Wernicke Area, and intraparietal sulcus. In Figure 6, two clusters of bins resulted in the highest accuracy. The first cluster included bins with short-range (10–25 mm) and medium-strength connections (0.3 < z < 0.5). The second cluster included bins with long-range (100–125 mm) and medium-strength connections (0.15 < z < 0.4).

**Figure 7:**
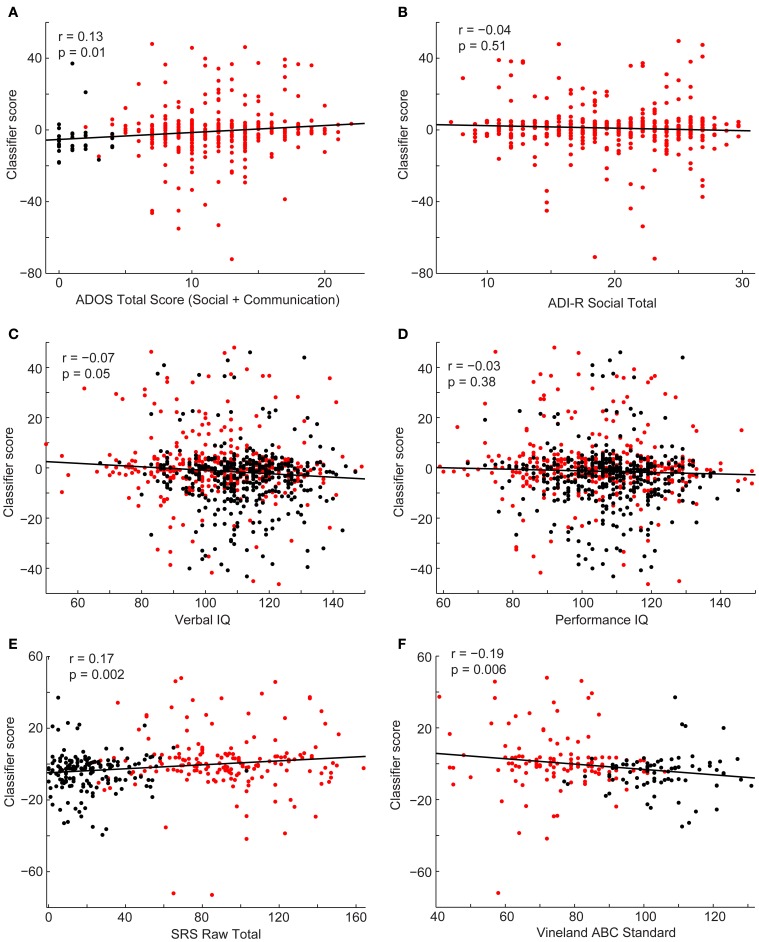
**Scatterplots depict the relationship between the classifier scores for control subjects (black) and subjects with autism (red) and the following behavioral measures: ADOS-G social + communication algorithm score (A), ADI-R social verbal algorithm score (B), verbal IQ (C), performance IQ (D), SRS total score (E), and Vineland Adaptive composite standard score (F)**. Correlation coefficients and corresponding *p*-values are included on the plots.

Finally, we investigated the relationship between the subject's classifier score and behavioral measures (Figure 7). Estimates of symptom severity (*r* = 0.13, *p* = 0.01), as measured by the ADOS social + communication algorithm score, and SRS (*r* = 0.17, *p* = 0.002) positively correlated with the classifier score, however, symptom severity, as measured by the ADI-R verbal domain algorithm score (*r* = −0.06, *p* = 0.30) or social domain algorithm score (*r* = −0.04, *p* = 0.51), and performance IQ (*r* = −0.03, *p* = 0.38) did not correlate with the classifier score. Verbal IQ (*r* = −0.07, *p* = 0.05) and Vineland adaptive behavior composite score(*r* = 0.17, *p* = 0.002) negatively correlate with the classifier score. In other words, as social function (lower SRS score is indicative of better social function), verbal IQ, and daily living skills increased and current level of symptom severity decreased, a subject was more likely to be classified as a control.

## Discussion

Functional connectivity MRI data from a set of 26.4 million “connections” per subject is able to successfully classify a subject as autistic or typically developing using a leave-one-out approach with an accuracy of 60.0% (*p* < 2.2 × 10^−10^), across a set of 964 subjects contributed from 16 different international sites. Overall specificity was 58.0% and overall sensitivity was 62.0%. Classification consisted of a weighted average of connections that used no information about the left out subject except for age, gender, site, and handedness. Using a weighted average of all 26.4 million connections resulted in a classification accuracy of 55.7% (*p* = 0.00017), with best accuracy (60.0%) achieved for a subset of connections that satisfied *p* < 10^−4^ for a difference between autism and control among remaining subjects for each left-out subject. Classification scores significantly covaried with metrics of current disease severity including ADOS-G (as opposed to ADI-R, which incorporates disease severity at early ages), SRS, and verbal IQ metrics. Classification accuracy significantly improved in sites for which longer BOLD imaging times were used, but no relationship was found between number of subjects contributed by a site and classification accuracy.

Classification accuracy was lower in this multisite study despite its much larger sample size when compared with a prior study using similar methods from a single site (Anderson et al., 2011d). The prior study achieved ~80% accuracy, with 90% accuracy for subjects under 20 years of age in both a primary cohort and a replication sample of affected and unaffected individuals from multiplex families. Several reasons may explain this difference. Expanding a classifier to accommodate multisite data necessarily involves dealing with many additional sources of variance. The pulse sequence, magnetic field strength, scanner type, patient cohort and recruitment procedures, scan instructions (eyes open vs. closed vs. fixation), BOLD imaging length, age distribution, gender differences, and population ethnicity all varied across sites. Each of these variables has the potential to decrease sensitivity and specificity of functional connectivity measurements for autism. Nevertheless, a multisite cohort helps test generalizability of the results across different samples, making it more likely that connections identified as discriminatory between autism and control reflect disease properties rather than particulars of a single dataset.

Classification accuracy in the multisite cohort varied with the subset of connections used to construct the classifier. This finding reflected a tradeoff between improved accuracy when using more connections with decreased accuracy when including less specific connections in the classifier. This result argues against a homogenous regional distribution of connectivity abnormalities in autism in favor of a heterogeneous spatial distribution of connectivity disturbances that involves specific brain regions. Analysis of brain regions most affected in abnormal connections herein confirms the findings of previous reports: areas of greatest abnormality included the insula, regions of the default mode network including posterior cingulate and medial prefrontal cortex, fusiform and parahippocampal gyri, Wernicke Area (posterior middle and superior temporal gyrus), and intraparietal sulcus (Anderson et al., 2011a,d; Gotts et al., 2012). All of these regions correspond to functional domains that are known to be impaired in autism, including attention, language, interoception, and memory. We note that some of these regions are in brain areas with relatively high susceptibility artifact and sensitivity to changes in brain shape (such as the medial prefrontal cortex). However, given the coherent distribution of the default mode network, we favor an interpretation of network-based differences attributable to autism rather than underlying structural or artifactual sources of these findings.

When interrogating subsets of connections from an independent dataset based on the Euclidean distance between ROIs and connection strength in a previous study, we found that the most informative connections consisted of typically strong connections between distant ROIs that were weaker in autism, and typically negatively correlated connections, that were less negative in autism (less anti-correlated) (Anderson et al., 2011d). In the current study, the connection bins based on strength and distance that showed greatest classification accuracy were not precisely the same connection bins found previously. Rather, they were adjacent to the bins in the previous study. This is the case because the classification algorithm in the current study takes advantage of larger numbers of connections. There was again a tradeoff between using more connections, given that individual connections exhibited relatively little information, and using sets of connections that differed more in autism. Thus, bins of medium strength connections (0.3 < *z* < 0.5) outperformed the more specific bins of stronger connections (*z* > 0.5) because the slightly weaker sets of connections included many more connections in the bin. This cautionary finding is relevant when attempting to identify the “optimal” set of connections for constructing candidate brain imaging biomarkers for ASD. Although specific affected regions appear to have autism connectivity abnormalities, classification schemes using only a small number of connections are likely to suffer from the high variance in metrics for individual connections.

This point is reinforced by a significant positive relationship between classification accuracy across sites and the length of BOLD imaging time per subject. Previous studies of test-retest reliability using functional connectivity MRI have shown that accuracy of results varies with one over the square root of BOLD imaging time (Van Dijk et al., 2010; Anderson et al., 2011c), with only moderate reproducibility when short BOLD imaging times such as 5 min are used (Shehzad et al., 2009; Van Dijk et al., 2010; Anderson et al., 2011c). This relationship would suggest that classifiers using information from many brain regions continue to show benefit from much longer imaging times, with continued improvements even after hours of imaging across multiple sessions per subject to the extent this is practical (Anderson et al., 2011c). Improvements in pulse sequence technology may also facilitate acquisition of greater numbers of volumes in shorter periods of time (Feinberg and Yacoub, 2012). The correlation between total imaging time and accuracy was more significant than the correlation between number of volumes used after scrubbing and accuracy. This might indicate that imaging time is more important than the number of volumes used. As multiband acquisition protocols become more prevalent (Setsompop et al., 2012), it will be important to determine the extent to which finer sampling vs. longer imaging time will contribute to specificity of BOLD fcMRI measurements.

In a prior study that examined the effect of BOLD imaging time on ability to identify functional connectivity values obtained from a single individual compared to a group mean, individual “connections” could only be reliably distinguished after 25 min of BOLD imaging time. The number of connections that could be reliably distinguished increased exponentially with imaging time for at least up to 10 h of total imaging time (Anderson et al., 2011c). Indeed, there is good theoretical basis that any desired accuracy can be obtained with sufficient imaging time, stretching into many hours. Although Van Dijk and colleagues report that the intrinsic connectivity measurements stabilize around 5 min of imaging time, they also state that noise continues to decrease at a rate of 1/sqrt(n), where n is the amount of imaging time (Van Dijk et al., 2010) (which is in accordance with our findings from (Anderson et al., 2011c). Moreover, they report that the stabilization is of composite network-level metrics rather than connections between small individual ROIs. In contrast, we have found that coarse network-level measurements are not particularly informative in classification compared to fine-grained metrics that take into account specific differences in the spatial distribution of connectivity. There may be no upper limit for continued improvements if more imaging time were obtained.

We found significant relationships between the classification score and some behavioral measures, such as social function and daily living skills, however, the proportion of variance in the behavioral measures that was explained by the linear relationship between the classification score and the behavioral measure was small (between 0.5 and 2.9%). This may be due to the overall poor accuracy of the classification approach. As accuracy and techniques for combining multisite data improves, we also expect an increase in the proportion of variance accounted for by the correlations.

Additional benefits may be achieved through improved classification algorithms that take advantage of machine learning techniques to allow more effective weighted combinations of connections. Similarly, multimodal classifiers remain a promising, relatively untapped method for characterizing diagnostic and prognostic information about autism. Given classification accuracies of single site datasets exceeding 80% for structural MRI (Ecker et al., 2010a,b; Jiao et al., 2010; Uddin et al., 2011; Calderoni et al., 2012; Sato et al., 2013), diffusion tensor MRI (Lange et al., 2010; Ingalhalikar et al., 2011), positron emission tomography (Duchesnay et al., 2011), and magnetoencephalography (Roberts et al., 2010, 2011; Tsiaras et al., 2011; Khan et al., 2013), it would be of great interest to determine whether different modalities identify similar cohorts of subjects correctly, and whether a combination neuroimaging approach that leverages these different features might be able to achieve even greater accuracy than any one alone.

Although multisite datasets such as those in ABIDE are invaluable for testing replicability of neuroimaging findings in autism, they contain inherent limitations that should be recognized. Large inhomogeneities in acquisition parameters, subject populations, and research protocols limit the sensitivity for detecting abnormalities. These inhomogeneities may overwhelm the ability of discriminating many findings, and may lead to overconfidence in a result as definitive because of the large sample of subjects used. There remains a need for replicating results in high-quality, carefully controlled individual datasets that may show increased sensitivity for some results compared to multisite data, as exhibited by classification accuracy in the present study. Preprocessing methods may also bias results in unpredictable ways, as has been suggested with head motion correction strategies (Power et al., 2012; Van Dijk et al., 2012) and regression procedures (Murphy et al., 2009; Anderson et al., 2011b; Saad et al., 2012). Datasets such as those in ABIDE will be of great value in testing multiple procedural manipulations in relatively large samples allowing determination of optimal processing methods for specific questions. Ultimately, it is unknown whether differences in resting state functional connectivity in autism arise from differential performance of the “resting” task or underlying differences in structural connectivity reflected in the measurements. Continuing comparison with structural metrics such as diffusion tensor imaging will help to clarify this point.

Nevertheless, it remains an attractive hypothesis that with longer imaging times, controlled acquisition strategies, integration of multimodal features, and improvement in classification methodology, neuroimaging may be able to contribute useful biological information to the clinical diagnosis and care of individuals with ASD and further elucidate pathophysiology and brain-based intermediate phenotypes.

### Conflict of interest statement

The authors declare that the research was conducted in the absence of any commercial or financial relationships that could be construed as a potential conflict of interest.
